# Comparison of the binding of the gastrin-releasing peptide receptor (GRP-R) antagonist ^68^Ga-RM2 and ^18^F-FDG in breast cancer samples

**DOI:** 10.1371/journal.pone.0210905

**Published:** 2019-01-15

**Authors:** Clément Morgat, Romain Schollhammer, Gaétan Macgrogan, Nicole Barthe, Valérie Vélasco, Delphine Vimont, Anne-Laure Cazeau, Philippe Fernandez, Elif Hindié

**Affiliations:** 1 Nuclear Medicine Department, University Hospital of Bordeaux, Bordeaux, France; 2 Univ. Bordeaux, INCIA, UMR-CNRS 5287, Talence, France; 3 CNRS, INCIA, UMR 5287, Talence, France; 4 Surgical Pathology Unit, Department of BioPathology, Institut Bergonié, Bordeaux, France; 5 INSERM, ACTION U1218, Bordeaux, France; 6 BioTis, INSERM U1026, Bordeaux, France; 7 Nuclear Medicine Department, Institut Bergonié, Bordeaux, France; University of South Alabama Mitchell Cancer Institute, UNITED STATES

## Abstract

The Gastrin-Releasing Peptide Receptor (GRPR) is over-expressed in estrogen receptor (ER) positive breast tumors and related metastatic lymph nodes offering the opportunity of imaging and therapy of luminal tumors. ^68^Ga-RM2 binding and ^18^F-FDG binding in tumoral zones were measured and compared using tissue micro-imaging with a beta imager on 14 breast cancer samples (10 primaries and 4 associated metastatic lymph nodes). Results were then assessed against ER expression, progesterone receptor (PR) expression, HER2 over-expression or not and Ki-67 expression. GRPR immunohistochemistry (IHC) was also performed on all samples. We also retrospectively compared ^68^Ga-RM2 and ^18^F-FDG bindings to ^18^F-FDG SUV_max_ on the pre-therapeutic PET/CT examination, if available. ^68^Ga-RM2 binding was significantly higher in tumors expressing GRPR on IHC than in GRPR-negative tumors (*P* = 0.022). In ER^+^ tumors, binding of ^68^Ga-RM2 was significantly higher than ^18^F-FDG (*P* = 0.015). In tumors with low Ki-67, ^68^Ga-RM2 binding was also significantly increased compared to ^18^F-FDG (*P* = 0.029). Overall, the binding of ^68^Ga-RM2 and ^18^F-FDG displayed an opposite pattern in tumor samples and ^68^Ga-RM2 binding was significantly higher in tumors that had low ^18^F-FDG binding (*P* = 0.021). This inverse correlation was also documented in the few patients in whom a ^18^F-FDG PET/CT examination before surgery was available. Findings from this in vitro study suggest that GRPR targeting can be an alternative to ^18^F-FDG imaging in ER^+^ breast tumors. Moreover, because GRPR antagonists can also be labeled with lutetium-177 this opens new avenues for targeted radionuclide therapy in the subset of patients with progressive metastatic disease following conventional treatments.

## Introduction

The Gastrin-Releasing Peptide Receptor (GRPR, also named BB2) is a G-protein coupled receptor of the bombesin family. Its over-expression on the membrane of tumor cells offers the opportunity of a selective targeting, using suitable radiolabelled bioconjugates, for positron emission tomography (PET) imaging and targeted radionuclide therapy (TRT). Tumors that can be targeted with GRPR-based radiotracers are notably, prostate cancer, breast cancer, lung cancer and colorectal cancer among others [1]. We have recently studied, using immunohistochemistry, the expression of GRPR ina large series of primary breast cancers and found that GRPR was overexpressed in 83.2% of ER-positive tumors but only in 12% of ER-negative tumors (p < 0.00001) [2]. When examined in molecular subtypes, GRPR is over-expressed in 86.2% of luminal-A and 82.8% of luminal-B HER2 negative tumors while triple negative breast cancers and HER2-enriched phenotypes exhibit GRPR over-expression in only 7.8% and 21.3% of cases. Importantly, lymph nodes metastases of GRPR-positive tumors also showed GRPR overexpression [2]. The association between GRPR and ER has also been documented at mRNA level by Dalm and colleagues [3]. Recently, GRP-R antagonists radiolabelled for PET imaging, demonstrated promising results in breast cancer patients. For example, in a small pilot study that used ^68^Ga-SB3, metastases were successfully visualized in 4 out of 6 patients [4]. In another study, ^68^Ga-RM2 could image with high contrast 13/18 primary breast tumors and detected metastatic lesions [5]. In a more recent study conducted in 34 women with suspected breast cancer, a novel GRPR antagonist, ^68^Ga-NOTA-RM26, was able to delineate primary breast tumors in 29/34 patients and lymph nodes metastases in 15/18 patients with node-positive disease [6]. Comparison of breast cancer imaging using GRP-R based radioantagonists and ^18^F-FDG is now needed to elucidate the place of GRP-R in the complex landscape of breast cancer imaging. This in vitro study aimed to assess the binding of ^18^F-FDG and that of the GRPR antagonist ^68^Ga-RM2 on representative breast cancer samples.

## Materials and methods

### Breast cancer samples

This study was approved by our institutional review board “Institut Bergonié Groupe Sein”. The project and data collection were performed according to the national French commission on informatics and liberty (CNIL). Prior to surgery, patients had given written consent to the use of part of the tumor material for research, after diagnostic procedures had been performed. Fourteen samples of formalin-fixed, paraffin-embedded breast cancer tissues (10 primary tumors and 4 associated metastatic lymph nodes) were retrospectively selected at Institut Bergonié. Sample characteristics’ are presented in Table 1. No patients had received neoadjuvant hormone therapy or chemotherapy. For each case, 6 successive slices were used: 1 for HES staining, 1 for GRP-R immunohistochemistry and 4 for micro-imaging of tissue radioactivity (one slice per radiopharmaceutical for total binding and one slice per radiopharmaceutical for non-specific binding). GRP-R immunohistochemistry was carried-out as previously described [2].

**Table 1:** Estrogen receptor (ER), progestin receptor (PR) expression, HER2 status, Ki-67 expression, molecular phenotypes and Gastrin-Releasing Peptide Receptor (GRP-R) expression in our series of samples.

*Sample*	*ER (%)*	*PR (%)*	*HER2 over-expression*	*Ki-67 (%)*	*Molecular phenotype*	*GRPR status*
Primary tumors						
1	70	90	No	2	Luminal-A	Pos
2	90	90	No	5	Luminal-A	Pos
3	80	30	No	20	Luminal-B	Pos
4	100	30	No	50	Luminal-B	Pos
5	90	0	No	30	Luminal-B	Neg
6	100	100	No	15	Luminal-B	Pos
7	0	0	Yes	20	HER2-enriched	Pos
8	0	0	Yes	35	HER2-enriched	Pos
9	0	0	Yes	25	Molecular apocrine	Pos
10	0	0	No	70	Basal	Neg
Metastatic lymph nodes						
11 from tumor 2	100	100	No	15	n.a. (not applicable)	Pos
12 from tumor 5	100	1	No	60	n.a.	Pos
13 from tumor 7	0	0	No	50	n.a.	Pos
14 from tumor 9	0	0	No	40	n.a.	Pos

#### Immunohistochemistry

IHC analyses were performed on 3μm tumor sections using specific antibodies directed against ER, PR, HER2/neu, Ki-67 and GRPR. All immunohistochemical techniques were performed on a Roche Ventana Benchmark ultra-automat. Details of antibody clones, manufacturers, dilutions used, incubation times, pretreatment buffers and staining kits are summarized in Table 2.

**Table 2:** Characteristics of antibodies used in this study.

Antibody	Clone	Supplier	Dilution	Incubation time	Unmasking	Revelation
ER	SP1	Roche Diagnostics (760–4605)	Ready to use	32 min	CC1 standard (64’)	UltraView Universal DAB
PR	1E2	Roche Diagnostics (790–4296)	Ready to use	12 min	CC1 short (36’)	UltraView Universal DAB
HER2	4B5	Roche Diagnostics (790–4493)	Ready to use	12 min	CC1 short (36’)	UltraView Universal DAB
Ki-67	30.9	Roche Diagnostics (790–4493)	Ready to use	32 min	CC1 standard (64’)	UltraView Universal DAB
GRP-R	polyclonal	Origene Technologies Rockville, Maryland	1/800	52 min	Protease 1 (4 min)	UltraView Universal DAB

Nuclear staining was assessed for ER and PR. A negative ER and/or PR status was defined by the presence of less than 1% of positive tumor cells. HER2/neu staining was scored according to the ASCO/CAP 2013 recommendations [7]. Ki-67 index was assessed semi-quantitatively and was considered low when 19% or less of tumor cell nuclei were stained and high when 20% or more tumor cell nuclei were stained. Molecular subtypes of breast cancers were derived from immunohistochemical markers (based on ER status, progesterone receptor PgR status, Ki-67 labeling index and HER2 status) according to St Gallen consensus [8] and Maisonneuve classification [9]. Molecular subtypes were defined as follows: Luminal A-like (HER2-, ER ≥ 1% and Ki-67 < 14% or Ki-67 ranging from 14% to 19% and PgR ≥ 20%); Luminal B-like HER2- (HER2-, ER ≥ 1% and Ki-67 ≥ 20% or Ki-67 14%–19% and PgR < 20%); Luminal B-like HER2+ (HER2+, ER ≥ 1%); HER-2 enriched (HER2+, ER = 0% and PgR = 0%); Triple-negative (ER = 0%, PgR = 0%, HER2-).

Results for GRP-R immunohistochemistry were expressed as previously described [2]. An experimented pathologist (GMG) quantified GRP-R expression and manually drew tumoral regions on the HES slice for quantification.

#### ^68^Ga-RM2 radiosynthesis and quality controls

Radiolabelling experiments were performed on an automated synthetisor (GE FastLab, GE Healthcare, GEMS Benelux, Belgium). Briefly, 40μg of RM2 (Life Molecular Imaging) was heated at 90°C during 5min using micro-waves with 1.1 mL ^68^GaCl_3_ (GalliEo generator with nominal activity of 1850 MBq, IRE Elit, Belgium) and 5mg of ascorbic acid. The raw solution was then purified on a C_18_ cartridge (WAT023501) preconditioned with 1mL ethanol (Merck) and 5 mL water (GE Healthcare). The final product was then eluted with 1 mL ethanol and formulated in PBS. ^68^Ga-RM2 was checked for radiochemical purity using HPLC (Phenomenex Luna C_18_; 250mm x 4.6mm x 5μm; 2.5 mL/min, λ = 220nm). The analytical HPLC system used was a JASCO system with ChromNAV software, a PU-2089 Plus quaternary gradient pump, a MD-2018 Plus photodiode array detector and Raytest Gabi Star detector. Amount of ^68^Ga-RM2 was determined by UV-HPLC by linear regression of the calibration curve established using the reference compound ^nat^Ga-RM2 (Life Molecular Imaging).

#### Tracer incubation and tissular micro-imaging

After dewaxing, rehydratation and unmasking, samples were pre-incubated during 10min at 37°C in Tris-HCl buffer at pH 7.4. Then, binding solution containing 5nM of ^68^Ga-RM2 or 1MBq (amount of ^18^F-FDG is not determined by the supplier) of ^18^F-FDG in Tris-HCl buffer pH 8.2, containing 1% of BSA (Sigma-Aldrich), 40μg/mL of bacitracin (Sigma-Aldrich) and 10nM of MgCl_2_ (Sigma-Aldrich) was applied. To assess non-specific binding, 1μM of reference compounds ^nat^Ga-RM2 or ^nat^F-FDG was added in adjacent slices. Samples were then incubated at 37°C for 2 hours. Afterwards, samples were rinsed 5 times during 8min in cold Tris-HCl buffer at pH 8.2 with 0.25% of BSA, 2 times during 8 minutes in cold Tris-HCl buffer at pH 8.2 without BSA and finally 2 times during 5 minutes in distilled water. Finally, samples were dried using air stream and were imaged using a beta imager 2000 (Biospace Lab).

#### Signal quantification

The M3Vision software was used for signal quantification. Total binding and non-specific binding were determined using the region of interest (ROI) method. First, a manual fusion by affine transformation of homologous structures was performed using the HES slice to match the radioactivity distribution to histology. Afterwards, on the total binding image (^68^Ga-RM2 or ^18^F-FDG alone) a first ROI (tumoral ROI) was placed on the tumoral zone and a second ROI (noise ROI), corresponding to noise, was placed around the tissue. Then, the same ROIs were applied on quantitative images from adjacent slices representing non-specific binding (^68^Ga-RM2 or ^18^F-FDG plus excess of reference compound) to define non-specific binding. Finally, data were exported on Excel software for processing. Parameters “Signal to Noise Ratio (SNR)” and “Delta” were then calculated. SNR was defined as the signal in tumoral ROI minus signal in noise ROI. Delta was calculated as follow:

$$Delta(\%)=\frac{SNRtotal\;binding-SNRnon\;specific\;binding}{SNRtotal\;binding}x\;100$$

#### Statistical analysis

Differences between mean values were assessed using non parametric t-test. A *P*-value of less than 0.05 was considered statistically significant. Statistical analysis was performed using GraphPad Prism software v 6.01.

#### Retrospective analysis of ^18^F-FDG PET/CT

We retrospectively analyzed pre-therapeutic ^18^F-FDG PET/CT performed at the Nuclear Medicine Department of Institut Bergonié. PET/CT had been performed before surgery in only 2 patients, corresponding to tissue samples 1 and 5 in Table 1. ^18^F-FDG uptake was measured as SUV_max_ in a VOI drawn on the breast tumor.

## Results

### ^68^Ga-RM2 radiosynthesis

^68^Ga-RM2 was obtained at a mean specific activity of 47.3 ± 16.7 GBq/μmol and a mean radiochemical purity of 99.52 ± 0.18% suitable for *in vitro* experiments.

### Comparison of ^68^Ga-RM2 binding and GRP-R immunohistochemistry

As a validation step we assessed whether tissular micro-imaging may accurately reflect IHC results. We stratified samples according to their GRP-R status determined by IHC and our results showed that the mean ^68^Ga-RM2 delta was significantly higher in GRP-R expressing tumors than in GRP-R-negative tumors (33.93 ± 17.55% vs 0.0 ± 0.0%; *P* = 0.022).

### ^68^Ga-RM2 and ^18^F-FDG bindings

#### Qualitative analysis

**Fig 1:**
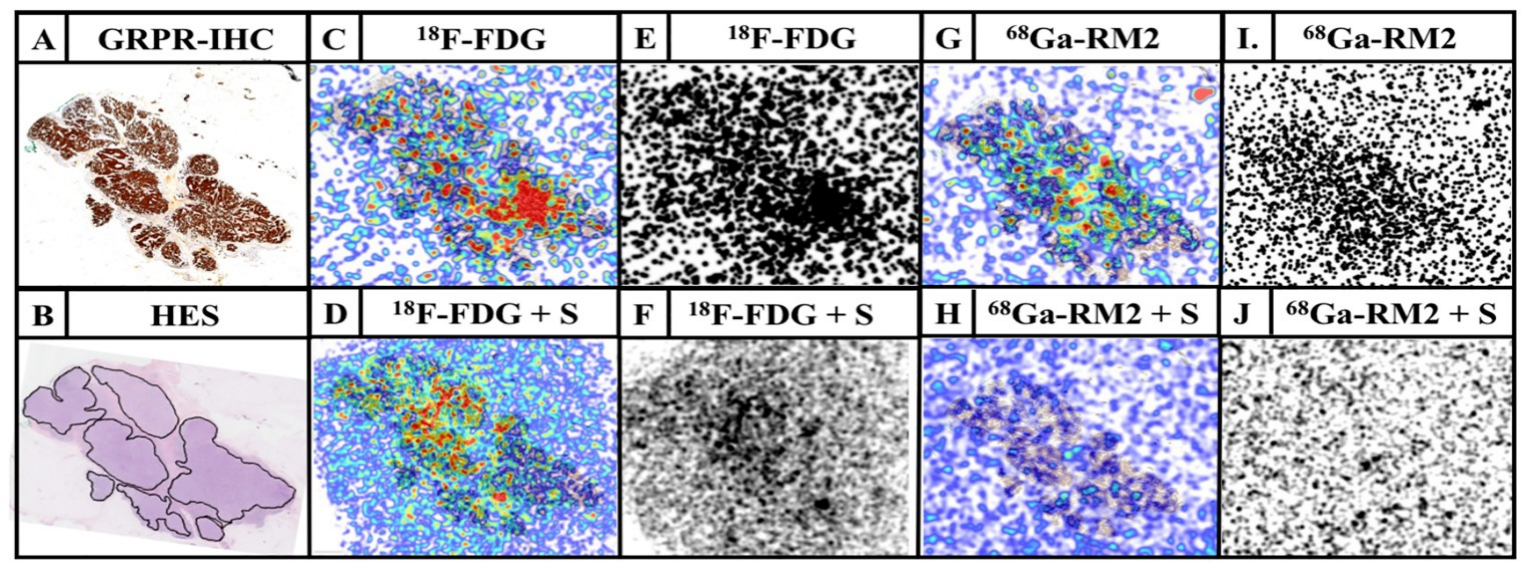
Example of a luminal B breast tumor sample. Representative GRP-R IHC (A; GRPR-IHC), HES staining (B; HES, black lines correspond to tumoral areas), ^18^F-FDG total binding fused with HES (C; ^18^F-FDG), ^18^F-FDG non-specific binding fused with HES (D; ^18^F-FDG + S), ^18^F-FDG total binding (E; ^18^F-FDG), ^18^F-FDG non-specific binding (F; ^18^F-FDG + S), ^68^Ga-RM2 total binding fused with HES (G; ^68^Ga-RM2), ^68^Ga-RM2 non-specific binding fused with HES (H; ^68^Ga-RM2 + S), ^68^Ga-RM2 total binding (I.; ^68^Ga-RM2), ^68^Ga-RM2 non-specific binding (J; ^68^Ga-RM2 + S). S refers to the reference compound used (^nat^F-FDG for ^18^F-FDG or ^nat^Ga-RM2 for ^68^Ga-RM2) to identify non-specific binding. In this sample, specific binding of ^68^Ga-RM2 is strong and evident while specific binding of ^18^F-FDG was overall weak and heterogeneous.

Qualitative analysis showed a good signal-to-noise ratio, and a binding in agreement with GRPR IHC with clear differences between total and non-specific bindings (Fig 1).

#### Quantitative analysis: Association between ^68^Ga-RM2 and ^18^F-FDG bindings and biological data

There was a significantly higher specific binding of ^68^Ga-RM2 in the ER^+^ group vs ER^-^ tumors (45.31 ± 13.23% vs 14.32 ± 9.20%; *P* = 0.030). Contrarily, there was a trend for lower ^18^F-FDG uptake in ER^+^ tumors (16.51 ± 28.45% vs 20.21 ± 17.77% *P* = 0.479).

There was also a higher specific binding of ^68^Ga-RM2 in the PR^+^ groups vs PR^-^ tumors (43.29 ± 13.24% vs 18.18 ± 18.43%; *P* = 0.028). Contrarily, ^18^F-FDG uptake looked similar in PR^+^ and PR^-^ tumors (21.70 ± 31.90% vs 21.13 ± 18.24%; *P* = 0.730).

A striking difference in ^68^Ga-RM2 binding was seen according to the percentage of Ki-67 staining. ^68^Ga-RM2 binding was significantly higher in the low Ki-67 group (49.24 ± 9.15% vs 20.62 ± 17.88%; *P* = 0.023). Contrarily so, there was a trend for higher ^18^F-FDG uptake in the high Ki-67 group vs low Ki-67 group (25.77 ± 26.43% vs 10.40 ± 12.35%; *P* = 0.287).

There were no significant differences in the HER2^+^ and HER2^-^ groups for ^68^Ga-RM2 or for ^18^F-FDG binding (Table 3).

**Table 3:** ^68^Ga-RM2 and ^18^F-FDG bindings in breast cancer samples according to biological data.

Biological data	*n*	^68^Ga-RM2	^18^F-FDG	*P-value*
*ER status*				
ER^+^ (≥ 10%)	8	45.31 ± 13.23%	16.51 ± 28.45%	**0.015**
ER^-^ (<10%)	6	14.32 ± 9.20%	20.21 ± 17.77%	0.483
***P-value***		**0.030**	0.479	
*PR status*				
PR^+^ (≥ 10%)	6	43.29 ± 13.24%	21.71 ± 31.90%	0.089
PR^-^ (<10%)	8	18.18 ± 18.43%	21.13 ± 18.24%	0.626
***P-value***		**0.028**	0.730	
*HER2 over-expression*				
Yes	3	16.13 ± 8.25%	30.19 ± 13.31%	0.200
No	11	32.25 ± 21.73%	18.98 ± 26.01%	0.163
***P-value***		0.280	0.269	
*Proliferation index Ki-67*				
High (≥20%)	10	20.62 ± 17.88%	25.77 ± 26.43%	0.783
Low (<20%)	4	49.24 ± 9.15%	10.40 ± 12.35%	**0.029**
***P-value***		**0.023**	0.287	

#### Quantitative analysis: Comparison of ^68^Ga-RM2 and ^18^F-FDG bindings

In ER^+^ tumors, binding of ^68^Ga-RM2 was largely higher than^18^F-FDG (45.31 ± 13.23% vs 16.51 ± 28.45%; *P* = 0.015), while in ER^-^ tumors binding of ^18^F-FDG was comparable to that of ^68^Ga-RM2 (*P* = 0.483). Therefore, the ratio of mean ^68^Ga-RM2 binding to ^18^F-FDG was 3.42 in ER^+^ tumors vs 0.71 in ER^-^ tumors. There was also a strong trend for higher ^68^Ga-RM2 binding than ^18^F-FDG in PR^+^ tumors (*P* = 0.089) while no differences were observed in the PR^-^ group (*P* = 0.626). In these subgroups, the ratio of mean ^68^Ga-RM2 binding to ^18^F-FDG was 1.99 in PR^+^ tumors vs 0.86 in PR^-^ tumors. In tumors with low Ki-67, ^68^Ga-RM2 binding was also significantly increased compared to ^18^F-FDG (49.24 ± 9.15% vs 10.40 ± 12.35%; *P* = 0.029). There was no differences in the bindings of ^68^Ga-RM2 and ^18^F-FDG in tumors with high Ki-67 (*P* = 0.783). These differences translate in a higher ratio of mean ^68^Ga-RM2 binding to ^18^F-FDG in low Ki-67 tumors (4.73 vs 0.80). In HER2- tumors, the ratio of mean ^68^Ga-RM2 binding to ^18^F-FDG was 1.70 while in HER2+ this ratio reaches only 0.53.

We also looked for ^68^Ga-RM2 binding in tumors considered negatives for ^18^F-FDG. Interestingly, ^68^Ga-RM2 binding was significantly higher in ^18^F-FDG-negative tumors: 36.03 ± 21.31% in ^18^F-FDG negative tumors vs 9.75 ± 11.06% in ^18^F-FDG-positive tumors, *P* = 0.021, S1 Fig.

### ^18^F-FDG PET/CT

Among patients studied using tissular micro-imaging, two had undergone ^18^F-FDG PET/CT imaging for staging before surgery (Table 4). The first patient had a low ^18^F-FDG uptake *in vivo* (SUV_max_ = 2.5), a negative ^18^F-FDG delta *ex vivo*, a high ^68^Ga-RM2 delta of 37.46% and a positive GRP-R IHC. The second patient had a high ^18^F-FDG uptake (SUV_max_ = 9.2), a high ^18^F-FDG delta of 42.97%, no ^68^Ga-RM2 binding and a negative GRP-R IHC.

**Table 4:** ^18^F-FDG SUV_max_ on PET/CT imaging in two breast cancer patients according to estrogen receptor (ER) expression, Ki-67 expression, ex vivo ^18^F-FDG and ^68^Ga-RM2 bindings and GRP-R immunohistochemistry.

*Patient*	*ER (%)*	*Ki-67 (%)*	*Molecular phenotype*	^*18*^*F-FDG SUV*_*max*_^†^	^*18*^*F-FDG delta (%)*^‡^	*GRP-R IHC*	^*68*^*Ga-RM2 delta (%)*^‡^
1	70	2	Luminal-A	2.5	0	Pos	37.46
2	90	30	Luminal-B	9.2	42.97	Neg	0

^***†***^: Data from ^18^F-FDG PET/CT

^***‡***^: Data from tissular micro-imaging experiments

## Discussion

The correlation between GRP-R overexpression in breast cancer and estrogen receptor positivity at protein level or mRNA level has been recently highlighted [2,3]. Moreover, it has been documented that when the breast primary is GRPR-positive, lymph node metastases also show GRPR overexpression [2,3]. Several clinical pilot studies have illustrated, *in vivo*, the potential of GRP-R for breast cancer imaging using radiolabelled GRP-R antagonists such as ^68^Ga-SB3, ^68^Ga-RM2 or ^68^Ga-NOTA-RM26 [4,5,6]. In some of these studies it was shown that ER-positive tumors can be visualized with high contrast [5,6]. ^18^F-FDG PET/CT is also a valuable tool for staging of invasive breast cancer [10]. Highly ^18^F-FDG-avid tumors are generally Elston and Ellis grade 3, have a high proliferation index and negative hormone receptor status, while somewhat lower uptake can be encountered in low grade ER-positive tumors and in lobular carcinoma [10]. Indeed, imaging ER-positive breast tumors, especially the luminal-A phenotype, might be challenging using ^18^F-FDG PET/CT in some patients [11]. Therefore, how GRP-R imaging would perform compared to ^18^F-FDG in ER-positive breast cancer deserves investigation. We aimed to compare on breast cancer samples the binding of a radiolabelled GRP-R antagonist, ^68^Ga-RM2, to that of ^18^F-FDG in order to better understand the potential of GRP-R imaging as a first step before a clinical study comparing the two tracers was launched.

Results of the present study on breast cancer samples showed that GRP-R targeting would be highly relevant in breast cancer, specifically in ER-positive tumors. Mean specific binding of ^68^Ga-RM2 was 45.31 ± 13.23% in ER-positive tumors and only 14.32 ± 9.20% in ER-negative tumors (*P* = 0.030). The opposite pattern was noted as regards ^18^F-FDG bindings. As a result, the ratio of mean ^68^Ga-RM2 binding to that of ^18^F-FDG binding in ER^+^ tumors was 3.42 vs 0.71 in ER^-^ tumors. Another important finding is the high ^68^Ga-RM2 binding in tumors with low Ki-67 (49.24 ± 9.15%) while tumors with high Ki-67 exhibited lower ^68^Ga-RM2 binding (20.62 ± 17.88%)(*P* = 0.023). Also, the ratio of mean ^68^Ga-RM2 to ^18^F-FDG binding in tumors with low Ki-67 was significantly higher than in tumors with high Ki-67 (4.73 vs 0.80). Overall, these results suggest a role for GRP-R PET imaging that could be complementary or superior to ^18^F-FDG imaging in ER-positive tumors with a low proliferation index.

Thus, ^18^F-FDG PET/CT and GRP-R imaging may be complimentary for imaging breast cancer and more specifically so the ER-positive subtypes. A study comparing a GRPR targeting radiotracer and ^18^F-FDG for primary staging or for restaging recurrent breast cancer would be appreciated. Another approach that could enhance tumor detection, is the possibility of a multiple targeting as demonstrated by ^68^Ga-BBN RGD that targets both GRP-R and integrin α_v_β_3_. In a pilot study, this heterodimeric radiopharmaceutical performed better than ^68^Ga-BBN (that targets only the GRP-R) in the detection of primary tumor and bone lesions in 11 patients [12]. Comparison with ^18^F-FDG would also be helpful for clinicians.

Finally, GRP-R targeting opens also attractive perspectives for radiopharmaceutical therapy of this subgroup of metastatic luminal patients with antagonists labelled with beta-emitters such as the lanthanides ^177^Lu [13] or ^161^Tb [14, 15] or with alpha emitters.

Limitations of this study, apart the number of samples and its retrospective nature, is the ^18^F-FDG tissular micro-imaging which may appear questionable. Cristallographic studies at the human glucose transporter 1 (GLUT-1) revealed that glucose uptake is a 2-step mechanism involving glucose binding before active transport [16]. Moreover, enhanced ^18^F-FDG uptake in tumors is not only related to overexpression of glucose transporters but also to enhanced hexokinase activity which was not assessed here. Therefore, our ^18^F-FDG tissular micro-imaging is relevant (clear displacement of ^18^F-FDG with reference compound) and revealed at least the ^18^F-FDG binding site but may over-estimate or underestimate ^18^F-FDG uptake.

In total, our data point that GRPR targeting should be helpful for imaging breast cancer and more specifically so the ER-positive subtypes. A study comparing a GRPR targeting radiotracer and ^18^F-FDG for primary staging and for restaging recurrent breast cancer is clearly needed.

## Supporting information

S1 Fig ^68^Ga-RM2 binding in ^18^F-FDG-positive (^18^F-FDG+) and ^18^F-FDG-negative (^18^F-FDG-) tumors. (TIF)
